# Cumulative Incidence in Monogenic Alzheimer’s Disease and Frontotemporal Dementia: Gene–Gene Interaction Effect

**DOI:** 10.3390/ijms27094081

**Published:** 2026-05-02

**Authors:** Andrea Geviti, Lorenzo Pagano, Mario Grassi, Claudia Saraceno, Alessandro Facconi, Silvia Fostinelli, Valentina Laganà, Giulia Giacomucci, Maria Sofia Cotelli, Valentina Cantoni, Assunta Ingannato, Silvia Bagnoli, Valentina Bessi, Antonio Longobardi, Alice Russotto, Sonia Bellini, Davide Lagrotteria, Ersilia Paparazzo, Giuliano Binetti, Alberto Montesanto, Benedetta Nacmias, Raffaele Maletta, Barbara Borroni, Roberta Ghidoni

**Affiliations:** 1 Service of Statistics, IRCCS Istituto Centro San Giovanni di Dio Fatebenefratelli, 25125 Brescia, Italy; ageviti@fatebenefratelli.eu (A.G.); mario.grassi@unipv.it (M.G.); 2 Molecular Markers Laboratory, IRCCS Istituto Centro San Giovanni di Dio Fatebenefratelli, 25125 Brescia, Italy; lorenzodaprato@hotmail.com (L.P.); csaraceno@fatebenefratelli.eu (C.S.); alessandro.facconi94@gmail.com (A.F.); alongobardi@fatebenefratelli.eu (A.L.); arussotto@fatebenefratelli.eu (A.R.); sbellini@fatebenefratelli.eu (S.B.); bborroni@fatebenefratelli.eu (B.B.); 3 Medical and Genomic Statistics Unit, Department of Brain and Behavioural Sciences, University of Pavia, 27100 Pavia, Italy; 4 MAC-Memory Clinic and Molecular Markers Laboratory, IRCCS Istituto Centro San Giovanni di Dio Fatebenefratelli, 25125 Brescia, Italy; sfostinelli@fatebenefratelli.eu (S.F.); gbinetti@fatebenefratelli.eu (G.B.); 5 Department of Primary Care, Regional Neurogenetic Centre (CRN), ASP Catanzaro, 88046 Lamezia Terme, Italy; valelagana@gmail.com (V.L.); raffaelegiovanni.maletta@asp.cz.it (R.M.); 6 Department of Neuroscience, Psychology, Drug Research and Child Health, University of Florence, 50139 Florence, Italy; giulia.giacomucci@unifi.it (G.G.); assunta.ingannato@unifi.it (A.I.); silvia.bagnoli@unifi.it (S.B.); benedetta.nacmias@unifi.it (B.N.); 7 Department of Continuity of Care and Frailty, ASST Spedali Civili di Brescia, 25123 Brescia, Italy; cotellim@gmail.com; 8 Department of Clinical and Experimental Sciences, University of Brescia, 25123 Brescia, Italy; vcantoni@fatebenefratelli.eu; 9 Neurology Unit, Dipartimento Neuromuscoloscheletrico E Degli Organi Di Senso, Careggi University Hospital Florence, 50139 Florence, Italy; 10 Department of Biology, Ecology and Earth Sciences, University of Calabria, 87036 Rende, Italy; davide.lagrotteria@unical.it (D.L.); ersilia.paparazzo@unical.it (E.P.); alberto.montesanto@unical.it (A.M.); 11 IRCCS Fondazione Don Carlo Gnocchi, 50143 Florence, Italy

**Keywords:** Alzheimer’s disease, frontotemporal dementia, cumulative incidence, gene–gene interaction, *APP*, *PSEN1*, *PSEN2*, *MAPT*, *GRN*, *C9orf72*

## Abstract

Monogenic forms of Alzheimer’s Disease (AD) and Frontotemporal Dementia (FTD) represent the two principal neurodegenerative disorders leading to early-onset dementia, primarily linked to mutations in key AD- and FTD-associated genes. The marked heterogeneity in age at onset and penetrance among carriers of pathogenic mutations suggests that monogenic variants act within a broader polygenic background. The combined impact of AD- and FTD-related genetic variation on disease incidence in monogenic forms remains largely unexplored. Herein, we investigate gene–gene interaction patterns in monogenic AD and FTD, with a focus on genetic variability in key AD (*APP*, *PSEN1*, *PSEN2*) and FTD (*MAPT*, *GRN*, *C9orf72*)-associated genes and their association with cumulative disease incidence. Within the GARDENIA Consortium, we studied 426 individuals from Italian pedigrees, including patients (n = 319) and presymptomatic (n = 107) carriers of causative variants in *APP* (n = 39), *PSEN1* (n = 71), *PSEN2* (n = 13), *MAPT* (n = 29), *GRN* (n = 188), and *C9orf72* (n = 86). Age at symptoms onset, age at last follow-up and sex were recorded. Whole exome sequencing was performed, focusing on non-causative variants (n = 64) in the key AD (*APP*, *PSEN1*, *PSEN2*) and FTD genes (*MAPT*, *GRN*, *C9orf72*). Weighted genetic burden scores were derived using Fine–Gray competing risk models to estimate variant-specific effects on cumulative AD and FTD incidence, accounting for mutually exclusive outcomes and family clustering. Model fit was evaluated using Akaike Information Criterion. Higher AD-risk-weighted burden scores in AD-related genes were associated with a significantly increased cumulative incidence of AD, while higher FTD-risk-weighted scores in FTD-related genes showed a trend toward association with increased cumulative incidence of FTD. A significant interaction between burden scores was observed. AD and FTD burden scores showed a negative interaction for AD (~79% attenuation) but a modest synergistic effect for FTD (~6% increase). These findings could imply context-dependent pleiotropy rather than simple additive genetic effects. Our study suggests that even in carriers oh highly penetrant AD or FTD causative variants, genetic background could substantially modulate cumulative disease incidence. Integrating polygenic information with monogenic status may improve prognostic stratification and inform precision approaches in dementia research and clinical trials.

## 1. Introduction

Alzheimer’s disease (AD) and frontotemporal dementia (FTD) represent the two principal neurodegenerative disorders leading to dementia. Although they differ in their clinical presentation and neuropathology, increasing evidence indicates substantial overlap in their underlying molecular and genetic mechanisms. Both diseases involve dysregulation of protein homeostasis, synaptic dysfunction, neuroinflammatory responses, and selective neuronal vulnerability. AD is widely considered to be a polygenic disorder resulting from complex interactions between environmental influences and multiple genetic risk factors [1,2]. The three common causative genes, *APP*, *PSEN1*, and *PSEN2*, associated with autosomal dominant early-onset AD (EOAD) [3,4], account for approximately 5–10% of EOAD cases with symptoms onset before the age of 65 years [4]. FTD represents the second most common cause of early-onset dementia, affecting individuals typically younger than 65 years [5,6]. The most common causative genes in familial monogenic FTD are *MAPT* [7,8], *GRN* [9,10], and *C9orf72* [11,12]. The OMIM phenotype codes associated with the diseases linked to the genes analyzed are summarized in Table S1.

While pathogenic mutations in *APP*, *PSEN1*, *PSEN2*, *MAPT*, *GRN*, and *C9orf72* genes can be sufficient to cause disease, mutation carriers exhibit marked heterogeneity in age at onset, penetrance, and clinical phenotype. Growing evidence suggests that genetic risk for neurodegeneration may be pleiotropic rather than additive, which in turn may modulate age at disease onset. Genetic evidence suggests shared susceptibility pathways: the *MAPT* H2 haplotype, for instance, is associated with reduced risk of late-onset AD, underscoring the role of tau regulation in both disorders [13,14]. Genetic studies have also shown that even within the same family, individuals carrying identical mutations may present different age at symptom onset. Such variability strongly suggests that monogenic mutations do not act in isolation, but rather in the context of a broader genetic background that modulates disease expression and trajectory [15,16,17,18,19]. Accumulating data from genome-wide association studies (GWAS) indicate that single nucleotide polymorphisms exert interactive effects on neurodegenerative disease risk. In AD, polygenic risk scores have been shown to influence disease susceptibility, age at onset, and neuropathological burden, as well as the rate of progression from mild cognitive impairment to dementia [20,21,22,23]. In contrast, the role of polygenic modifiers in FTD remains less well characterized, despite well-established incomplete penetrance and molecular heterogeneity among carriers of identical pathogenic variants, both within and across families. Data from a GWAS revealed an additive effect of genetic variants and their implication in modulating disease onset in Italian sporadic FTD patients [24].

To the best of our knowledge, no studies have investigated the combined effect of genetic variants in *APP*, *PSEN1*, *PSEN2*, *MAPT*, *GRN*, and *C9orf72* on cumulative disease incidence in monogenic AD and FTD.

Herein, we investigate how gene–gene interaction shape disease risk in monogenic AD and FTD, with a focus on genetic variability in key AD (*APP*, *PSEN1*, *PSEN2*) and FTD (*MAPT*, *GRN*, *C9orf72*)-associated genes and their impact on cumulative disease incidence.

## 2. Results

Demographic and clinical characteristics of the 426 subjects, stratified by diagnosis (AD, FTD or presymptomatic) and assigned genetic group are shown in Table 1 and Table 2. The complete list of *APP*, *PSEN1*, *PSEN2*, *MAPT*, *GRN*, and *C9orf72* pathogenic/VUS variants as well as the descriptives of the non-causative variants identified by Whole Exome Sequencing (WES) for each of the genetic groups of interest are shown in Supplementary Tables S2 and S3, respectively. A distribution of the non-causative variants identified by WES is shown in Supplementary Figure S1.

### 2.1. Time-to-Onset Analysis

**Figure 1:**
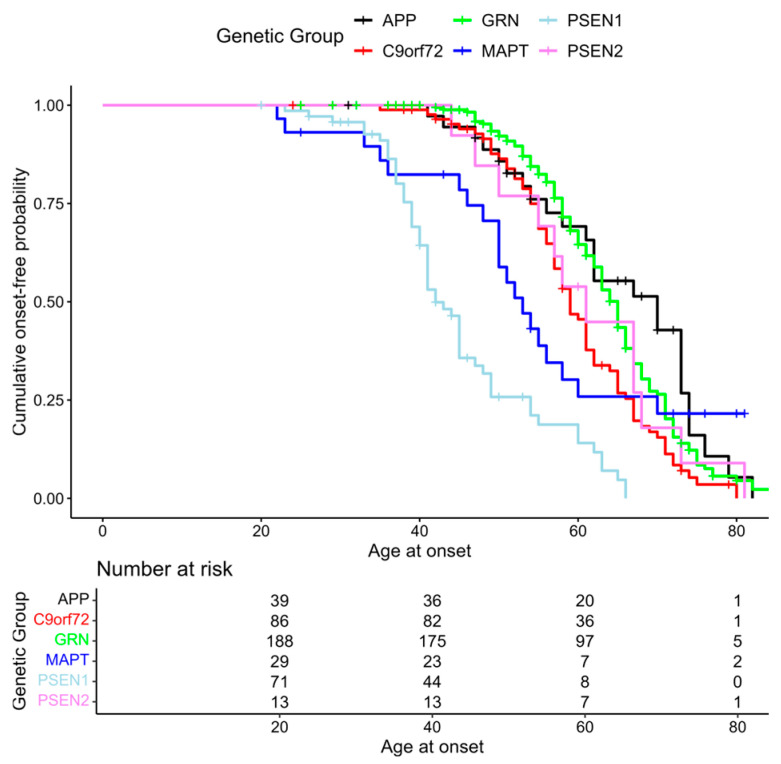
Composite endpoint Kaplan–Meier curves stratified for the genetic groups of interest.

The role of genetic factors was explored through Kaplan–Meier curves, comparing groups in terms of onset probability. As illustrated in Figure 1, the curves for the composite endpoint configuration show an overall significant difference between the genetic groups (log-rank *p* < 0.001). Carriers of the *PSEN1* causative variants exhibited a higher early-onset risk, whereas *APP* and *GRN* carriers showed a relatively lower early-onset risk.

**Figure 2:**
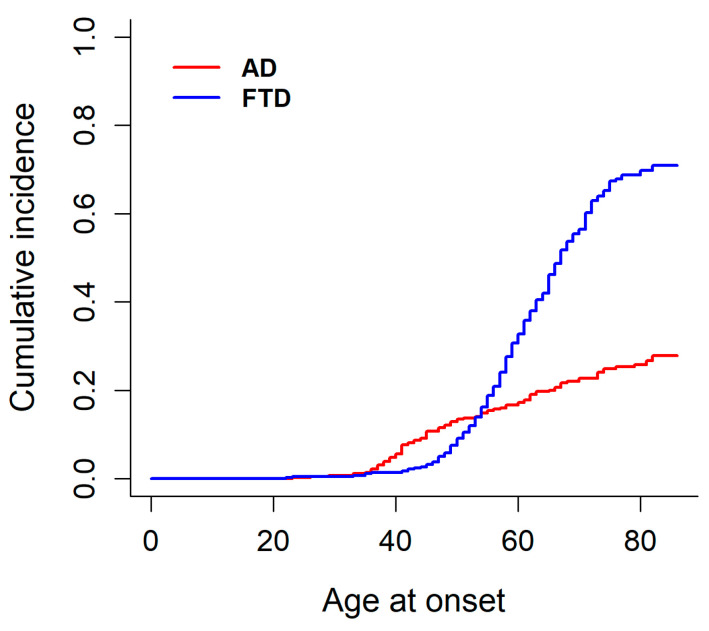
Cumulative incidence functions for AD and FTD.

The CIFs for AD and FTD (Figure 2) illustrates that the probability of developing AD is initially higher than that of FTD up to the mid-50s, after which the risk of FTD increases sharply and surpasses that of AD, while the incidence of AD continues to grow but at a slower rate.

### 2.2. Genetic Scores

When deriving the non-causative variant weights used for the genetic burden scores, the Fine–Gray model did not return an estimable regression coefficient for some of the variants, likely due to rare genotype frequencies (recessive homozygotes = 0 or heterozygotes = 1 or vice versa). In these cases, no β was produced and the weight was conservatively set to zero. LD was evaluated by computing pairwise r^2^ values from the genotype data and visualizing them using an r^2^-based heatmap (Figure S1). This analysis identified a clear LD block involving six *PSEN1* variants, while all other variants showed little evidence of LD. Because the variant weights were obtained from univariate Fine–Gray models, each variant analyzed independently, the six *PSEN1* variants were entered into the model as a single unweighted sum score. Moreover, no correlation was observed between the non-causative variants identified by WES and the excluded pathogenic variants and VUS, indicating that the variants contributing to the burden scores were genetically independent from known high-penetrance mutations.

For the CIFs stratified by AD and FTD scores, the four genetic risk scores were dichotomized at the median, creating groups of low (0) versus high (1) scores (i.e., AD low and AD high; FTD low and FTD high). Among the AD subjects, only ~9% had high AD scores. In contrast, FTD subjects showed a more balanced distribution between low and high FTD scores (~53% > median), suggesting that multiple FTD loci contribute more heterogeneously to cumulative disease incidence.

### 2.3. Interaction Analysis

**Figure 3:**
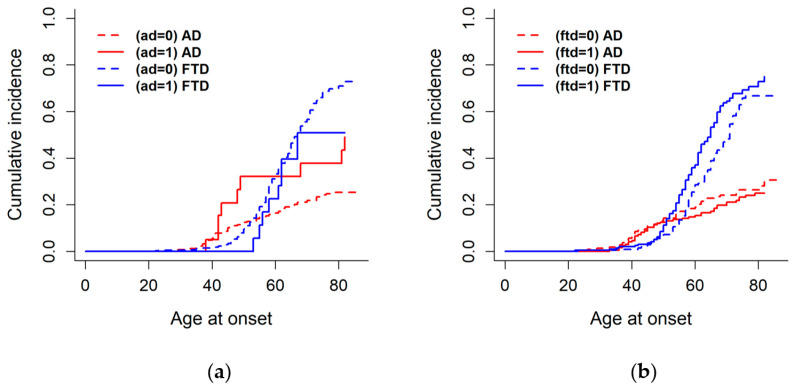
Stratified cumulative incidence functions showing the interaction between AD and FTD genetic scores and diagnostic group in cumulative onset probability. (**a**) Individuals with high AD genetic scores (above median; *APP*, *PSEN1*, *PSEN2*) show higher cumulative AD incidence and a non-significant lower FTD incidence. (**b**) Individuals with high FTD genetic scores (above median; *MAPT*, *GRN*, *C9orf72*) show higher cumulative FTD incidence and a non-significant lower AD incidence.

In Figure 3 are displayed the CIFs for the stratifications that yielded significant differences, specifically, the cumulative incidence of AD stratified by AD genetic score (below vs. above the median), and the cumulative incidence of FTD stratified by FTD genetic score (below vs. above the median). In panel (a), individuals with a high AD (above the median) genetic score, reflecting a higher weighted burden non-causative of variants in the three AD-causative genes (*APP*, *PSEN1*, *PSEN2*), showed a significantly higher cumulative incidence of AD (Gray’s test, *p* = 0.041) and a lower, although non-significant, cumulative incidence of FTD (*p* = 0.194). Similarly, in panel (b), individuals with a high FTD (above the median) genetic score, reflecting a higher weighted burden of non-causative variants in FTD-causative genes (*MAPT*, *GRN*, *C9orf72*), exhibited a significantly higher cumulative incidence of FTD (*p* = 0.022) and a lower, non-significant cumulative incidence of AD (*p* = 0.414).

To confirm the gene–gene interaction effect, the combined effects of the continuous AD and FTD scores on cumulative disease incidence were tested with Fine–Gray’s competing risk models under seven different specifications (see Section 4.4), across two scenarios (treating AD and FTD as the primary events in turn, with the other event considered as a competing risk). Model selection based on AIC identified the best-fitting model in each case. Because AD and FTD scores exhibited high variability that could lead to inflated hazard ratios in Fine–Gray models, they were standardized using the interquartile range between the 20th and 80th percentiles. Finally, the scores included in these models were further validated using the 5-fold cross-validation procedure.

#### 2.3.1. AD Onset

Considering AD onset as the event of interest, analyses using the raw AD and FTD scores indicated that models including only AD and FTD scores, as well as the model containing AD, FTD, and their interaction (AD+FTD+AD×FTD), yielded the lowest AIC values. We focused on the interaction model because the interaction term was statistically significant and its AIC was comparable to the simpler models (see Supplementary Table S4). Results obtained with the raw scores are reported in Supplementary Table S4.

Using the cross-validated scores, the model including AD, FTD, and their interaction (AD+FTD+AD×FTD) provided the lowest AIC among the tested models. In this model, when the FTD score is at its mean value (FTD = 0 after standardization), subjects above the 80th percentile of the AD score distribution have a 2.49-fold higher hazard of developing AD compared to subjects below the 20th percentile (HR = 2.49, 95% CI 1.42–4.35, *p* = 0.0014). This means that over the observed age range, individuals with higher AD genetic burden show an overall higher probability of experiencing AD onset, accounting for the competing risk of developing FTD. Similarly, when the AD score is at its mean (AD = 0), subjects above the 80th percentile of the FTD score have a 1.82-fold higher hazard of developing AD (HR = 1.82, 95% CI 1.16–2.87, *p* = 0.0093). The interaction term was also significant (HR = 0.21, 95% CI 0.09–0.052, *p* = 0.0007), indicating that the combined increase in AD and FTD scores reduces the hazard of AD (−79%) compared to what would be expected from the multiplication of the individual effects alone. Results of the Fine–Gray model using 5-fold cross-validated scores were consistent with those obtained using the raw scores and reported in Table 3 (see Supplementary Table S5 for AIC values).

#### 2.3.2. FTD Onset

Considering FTD onset as the event of interest, analyses using the raw AD and FTD scores indicated that the models including only the FTD score, the model with both AD and FTD scores, and the full interaction model (AD+FTD+AD×FTD) all achieved comparably low AIC values (see Supplementary Table S4). As before, we retained the latter because the interaction was significant. Results obtained with the raw scores are reported in Supplementary Table S4.

Using the cross-validated scores, the interaction term in the full model did not reach statistical significance, while the model including AD and FTD scores (AD+FTD) provided the AIC among the tested models. In this model, when the FTD score is at its mean (FTD = 0), subjects above the 80th percentile of the AD score distribution showed a trend toward a higher hazard of developing FTD compared to subjects below the 20th percentile (HR = 1.49, 95% CI 0.997–2.21, *p* = 0.0515). Similarly, when the AD score is at its mean (AD = 0), subjects above the 80th percentile of the FTD score have a 1.25-fold higher hazard of developing FTD (HR = 1.25, 95% CI 1.22–1.43, *p* = 3.94 × 10^−12^). The interaction term was also significant (HR = 1.06, 95% CI 1.07–1.46, *p* = 0.0051). Results of the Fine–Gray models with 5-fold cross-validated are reported in Table 3 (see Supplementary Table S5 for AIC values).

#### 2.3.3. Sensitive Analysis Excluding APOE ε4 Carriers

As an additional robustness analysis, we re-estimated the models after excluding carriers of the *APOE* ε4 allele. Of the total cohort, n = 79 individuals carried at least one ε4 allele, and the analysis was therefore conducted in the remaining n = 347 participants. The results were consistent with those obtained in the full sample for the analysis with the raw scores: both genetic scores remained significantly associated with disease onset, and their interaction term also remained statistically significant in both the AD and FTD models (AD interaction HR = 0.71, 95% CI 0.57–0.88, *p* = 0.002; FTD interaction HR = 1.06, 95% CI 1.01–1.11, *p* = 0.029). Also, analyses based on the cross-validated AD and FTD scores showed patterns broadly consistent with those observed in the main models. For AD onset, the interaction term between AD and FTD scores remained statistically significant (HR = 0.45, 95% CI 0.21–0.94, *p* = 0.033), supporting the presence of a negative interaction pattern even after excluding ε4 carriers. For FTD onset, the interaction term in the full model was not significant, as in the full sample analysis. Overall, these results indicate that the main patterns observed in the primary analyses are not driven by APOE ε4 carrier status.

## 3. Discussion

In this study we investigated how non-causative genetic variants in key AD-related genes (*APP*, *PSEN1*, *PSEN2*) and FTD-related genes (*MAPT*, *GRN*, *C9orf72*) influence cumulative incidence of autosomal dominant forms of AD and FTD, using event-specific weighted genetic burden scores within a Fine–Gray competing risk framework. Importantly, although the same set of variants entered the models, variant weights were estimated separately for AD and FTD incidence. Thus, each burden score reflects the cumulative contribution of variants in AD or FTD-associated genes to the cumulative incidence of a specific clinical outcome.

We clarify that our analytical framework should be interpreted as inferential rather than predictive. The aim of the present study was to investigate associations and interaction patterns within the observed cohort in order to generate biological and mechanistic insights, rather than to build a model optimized for predicting disease onset in new individuals. In statistical methodology, inferential models focus on estimating relationships among variables within the available data, whereas predictive models aim to maximize out-of-sample prediction accuracy and therefore require explicit validation procedures. Within this inferential framework, the goal is therefore to characterize potential interaction mechanisms among genetic factors rather than to derive prediction tools. However, we performed and presented results obtained with internally cross-validated gene scores to add robustness to our findings.

Our study suggests that, even in carriers of highly penetrant AD or FTD causative variants, genetic background may contribute to modulating cumulative disease incidence. In our cohort, higher AD-risk-weighted scores derived from AD-associated genes were associated with an increased cumulative incidence of AD, while higher FTD-risk-weighted score derived from FTD-associated genes showed a trend toward a higher hazard of developing FTD. Individually, these two scores behaved as expected. A higher AD-risk-weighted burden of AD-associated genes variants significantly hastened the cumulative AD onset probability, mirroring prior work showing that aggregating common AD loci stratifies disease risk and timing, and is associated with faster conversion from mild cognitive impairment to greater AD neuropathology [23]. Similarly, a higher FTD-risk-weighted burden of FTD-associated genes variants increased the cumulative incidence of FTD, albeit with a smaller effect size than observed for AD and showing only a trend. Interestingly, we observed evidence of an interaction between AD- and FTD-derived burden when AD was the event of interest. The interaction between the AD-risk-weighted burden of AD-associated genes and the AD-risk-weighted burden of FTD-associated genes was negative, corresponding to a ~79% attenuation of the AD cumulative incidence.

Our results are compatible with the possibility of antagonistic pleiotropy: certain alleles (or combinations) may predispose to one form of dementia at the expense of another. Biologically, these interactions may reflect competing pathogenic pathways. Genes such as *MAPT* and *APOE* illustrate this complexity. For instance, the *MAPT* H1 haplotype is a known risk factor for FTD but has also been implicated in AD [25,26,27]. In contrast, *APOE* alleles show nearly opposite effects: the ε4 allele strongly increases AD risk, but ε2, which is AD-protective, appears to increase FTD risk. Notably, Verpillat et al. reported *APOE* ε2 was enriched in FTD patients (OR ≈ 2.0) despite being the lowest-risk allele in AD [28]. This exemplifies true antagonistic pleiotropy: the same variant confers protection against one disease while increasing risk for another. In our cohort, even after excluding *APOE* ε4 carriers, the interaction term (x:z) remained present and statistically significant in the AD model. These findings suggest that the observed interaction is unlikely to be solely driven by the presence of *APOE* ε4 carriers. The genetic interaction we observed could have clinical and biological implications. Shared cellular pathways (e.g., synaptic function, inflammation, protein homeostasis) may be tuned differently by different sets of risk alleles.

Clinically, our findings raise the possibility that integrating polygenic scores with monogenic status could help refine the characterization of disease onset and subtype of dementia. This could inform monitoring strategies and trial design.

Nevertheless, these results should be interpreted with caution. Our sample, though enriched for pathogenic rare variants carriers, remains relatively modest for interaction testing; larger studies will be needed to confirm the antagonistic effect on AD risk. Moreover, low variability of AD-associated gene burden scores may limit the precision and stability of interaction effect estimates, and therefore warrant cautious interpretation of their magnitude.

In this context, the observed patterns should be interpreted as preliminary signals of the underlying genetic architecture in this cohort rather than definitive estimates of interaction effects. Although detectable within the present dataset, their generalizability cannot be assumed without replication in independent cohorts. Accordingly, these findings are best viewed as exploratory, hypothesis-generating evidence highlighting potentially informative relationships between AD- and FTD-related genetic backgrounds.

We also limited weighted genetic burden scores to variants in known AD/FTD genes. The gene selection was guided by predefined biological hypotheses and focused specifically on genes with strong, well-established evidence of causing disease through monogenic mechanisms: *APP*, *PSEN1*, *PSEN2*, *MAPT*, *GRN*, and *C9orf72*. Extending the analysis to other genes or, possibly, the entire genome could modify or even reverse the interactions we see. Finally, our cohort was largely of European ancestry. Generalizing our results to other populations, given different allele frequencies and linkage patterns, will require further studies.

Future work should extend these observations in several directions. First, larger multi-cohort analyses are needed, ideally with whole-genome burden scores for both diseases. A genome-wide burden scores could capture unknown pleiotropic loci and better quantify antagonistic effects. Second, mechanistic research should probe how specific pathways mediate the genetic interactions. Moreover, future mechanistic studies should investigate the structural and functional consequences of individual variants, including their localization within functional protein domains, as this may help elucidate the molecular basis of the antagonistic and synergistic interactions observed here. Finally, exploring these interactions in sporadic AD/FTD could reveal whether similar antagonistic relationships operate more broadly.

## 4. Materials and Methods

### 4.1. Participants

This study was conducted within the framework of the GARDENIA project (“Genetic and epigenetic modulAtors in Rare neurodegenerative disease with DEmentia: a National study on autosomal dominant Alzheimer disease and genetic frontotemporal degeneration with dementIA”), funded by the European Union—Next Generation EU (PNRR-MR1-2022-12375654). For the purposes of the present study, we included patients with AD (n = 93) [29,30], or presymptomatic subjects (n = 30) carrying causative variants in the *APP*, *PSEN1* or *PSEN2* genes, and patients with FTD (n = 226) [31,32,33] or presymptomatic subjects (n = 77) carrying causative variants in the *MAPT*, *GRN* or *C9orf72* genes. Participants were drawn from multiple families, with several individuals belonging to the same family resulting in several family clusters. The pathogenicity of genetic variants was assessed according to the variant interpretation criteria established by the American College of Medical Genetics and Genomics (ACMG) [34]. Clinical significance data were retrieved from ClinVar (https://www.ncbi.nlm.nih.gov/clinvar/ (accessed on 15 April 2026)), VarSome (https://varsome.com/ (accessed on 15 April 2026)) [35], and the Human Gene Mutation Database (HGMD) (https://www.hgmd.cf.ac.uk/ac/index.php (accessed on 15 April 2026)) [36]. Additional information on mutations associated with AD and FTD was obtained from Alzforum (https://www.alzforum.org/ (accessed on 15 April 2026)), FTDtalk (https://www.ftdtalk.org/ (accessed on 15 April 2026)), and Genetic FTD Initiative database (GENFI; https://www.genfi.org/ftd-mutation-database/ (accessed on 15 April 2026)). In addition to pathogenic variants, the cohort also included subjects carrying variants of uncertain significance (VUS), which are currently under ongoing evaluation and follow-up by the research centers. The complete list of *APP*, *PSEN1*, *PSEN2*, *MAPT*, *GRN*, and *C9orf72* causative variants are shown in Supplementary Table S2. All databases were accessed on 16 December 2025. Demographic characteristics were recorded. Age at symptom onset was defined as the year in which one of the core symptoms of AD or FTD was first noted by the patient, family member, or health care provider or allied health professional as recorded in the medical record. Presymptomatic individuals were defined as carriers of pathogenic variants or VUS in AD- or FTD-related genes who had not yet developed clinical symptoms at last follow-up. Age in presymptomatic individuals was defined as the age at last follow-up.

DNA samples belonging to Italian pedigrees, were already stored at the institutional biobank/biorepositories of the three Italian centers belonging to GARDENIA Consortium: IRCCS Centro San Giovanni di Dio Fatebenefratelli BioBank Brescia, Italy (bbmri-eric ID: IT_138442378660827 and Orphanet Biobank) (n = 250); Azienda Ospedaliero Universitaria Careggi in Florence, Tuscany (Orphanet Biorepository of neurodegenerative disorders) (n = 57); Azienda Sanitaria Provinciale di Catanzaro, Calabria (Orphanet Biobank CRN (Regional Neurogenetic Centre)) (n = 119). Informed consent was obtained from all subjects involved in the study. The study was conducted in accordance with the Declaration of Helsinki, and approved by the local Ethics Committee “Comitato Etico IRCCS San Giovanni di Dio Fatebenefratelli” of the IRCCS Centro San Giovanni di Dio Fatebenefratelli, Brescia (Prot. N. 63/2022; date of approval: 7 December 2022).

### 4.2. Genetic Analysis

Whole exome sequencing was performed using bead-linked transposome and hybrid-capture chemistry on Illumina NextSeq 2000 system (Illumina, San Diego, CA, USA). The quality assessment of gDNA was performed on a 0.8% agarose gel, and gDNA was quantified with a Qubit dsDNA BR Assay Kit (Thermo Fisher Scientific, Waltham, MA, USA). A total of 250 ng of gDNA was used for library preparation with Illumina DNA Prep with Exome 2.5 Enrichment kit (Illumina, San Diego, CA, USA). gDNA was tagmented, amplified, and purified with Illumina Purification Beads (Illumina, San Diego, CA, USA). The size, quality, and quantity of libraries were assessed with a High Sensitivity DNA kit on a Bioanalyzer instrument (Agilent Technologies, Santa Clara, CA, USA). A 2000 pM sample of the pooled library was loaded with NextSeq 1000/2000 P3 reagents (Illumina, San Diego, CA, USA) and sequenced on an Illumina NextSeq 2000 system (Illumina, San Diego, CA, USA).

### 4.3. Bioinformatic Analysis: Data Pre-Processing, Mapping, and Variant Calling

Exome sequencing data were first subjected to quality assessment with FastQC (version 0.12.1) [http://www.bioinformatics.babraham.ac.uk/projects/fastqc/]. Adapters and low-quality reads were filtered out using Trimmomatic (version 0.39) [37], while the remaining high-quality reads were mapped to the GRCh38/hg38 reference genome employing BWA-MEM (version 0.7.17-r1188) [38]. Duplicate reads were flagged with the MarkDuplicates module from Picard (version 3.0) [https://broadinstitute.github.io/picard/]. Base quality recalibration was carried out with BaseRecalibrator and ApplyBQSR modules from GATK (version 4.4.0.0) [https://gatk.broadinstitute.org/]. Afterwards, variant calling was performed via the HaplotypeCaller module (GATK, version 4.4.0.0), producing gVCF files that were later combined using GenotypeGVCFs (GATK, version 4.4.0.0). To reduce false positives, Variant Quality Score Recalibration (VQSR) was applied through the VariantRecalibrator and ApplyVQSR modules (GATK, version 4.4.0.0). On average, sequencing depth per sample reached 120X, with more than 90% of samples achieving a coverage threshold ≥ 20X in *APP*, *PSEN1*, *PSEN2*, *MAPT*, *GRN*, and *C9orf72* genes. Functional interpretation of variants was conducted with ANNOVAR (2020-06-08 release) [39]. We focused on non-synonymous variants, including missense, stop-gain, stop-loss, start-loss, and frameshift indels, considering both common and rare variants. The analysis was restricted to genes known to be causative for AD (*APP*, *PSEN1*, *PSEN2*) and FTD (*MAPT*, *GRN*, *C9orf72*).

### 4.4. Statistical Analysis

Descriptive statistics were computed to characterize the study sample. Continuous variables were summarized as mean and standard deviation (mean, SD), and categorical variables as counts and percentages (N, %). Group comparisons for continuous variables were performed using *t*-tests or analysis of variance (ANOVA). Differences in categorical variables were assessed using chi-squared tests.

#### 4.4.1. Time-to-Onset Analysis

Time-to-event analysis was performed using age at symptom onset for affected individuals, and age at last follow-up for presymptomatic participants. Kaplan–Meier curves [40] were generated stratifying participants by genetic groups, providing a descriptive comparison of age-at-onset distributions between them. The composite endpoint was considered: presymptomatic subjects were censored, and the event (1 = yes, 0 = no) was defined as the first clinical onset of either AD or FTD, regardless of disease subtype. Each variant was individually tested for its association with the cumulative incidence of AD and FTD (based on age at onset) using a Fine–Gray competing risk regression model [41], with AD or FTD specified as the event of interest in separate analyses. This model is useful to investigate the cumulative probability of disease onset while accounting for mutually exclusive outcomes. In this framework, AD and FTD were considered as competing events. The model estimates subdistribution hazard ratios (sHRs), which quantify the relative effect of covariates (in this case, disease-specific scores) on the cumulative incidence of one event type, while explicitly accounting for the fact that individuals may alternatively experience the other event. In practice, this approach allows the interpretation of results in terms of how genetic variation modifies the overall probability of developing AD given that FTD may also occur (and vice versa). The models were adjusted for family clustering (see Section 4.1).

#### 4.4.2. Genetic Scores

We derived weighted genetic burden scores reflecting each subject’s cumulative load of variants in AD-related and FTD-related genes. From Fine–Gray models, the marginal (univariate) regression coefficient (β) was extracted and used as the weight for that variant. Specifically, for each subject, two weighted burden scores were then calculated as: $S_{i}^{\left(AD\right)}=\sum_{j=1}^{m}G_{ij}\cdot \beta_{j}^{\left(AD\right)}$, and $S_{i}^{\left(FTD\right)}=\sum_{j=1}^{m}G_{ij}\cdot \beta_{j}^{\left(FTD\right)}$, where $G_{ij}$ is the genotype for subject i at variant j (coded as 0, 1, or 2 copies of the minor allele), and $\beta_{j}^{\left(AD\right)}$ and $\beta_{j}^{\left(FTD\right)}$ are the Fine–Gray marginal regression coefficients for that variant in the given model AD = (*APP*, *PSEN1*, *PSEN2*) and FTD = (*MAPT*, *GRN*, *C9orf72*). Because each variant received two different weights (one from the AD model and one from the FTD model), and because we also separated variants based on whether they belonged to AD-related genes (*PSEN1*, *PSEN2*, *APP*) or FTD-related genes (*MAPT*, *GRN*, *C9orf72*), this procedure yielded four burden scores for each subject: (i) AD-risk-weighted AD variants score, (ii) AD-risk-weighted FTD variants score, (iii) FTD-risk-weighted AD variants score and (iv) FTD-risk-weighted FTD variants score. Known pathogenic variants and VUS were excluded from the burden-score analyses and only non-causative variants (WES-identified variants not considered the primary pathogenic drivers in our cohort) were retained (64 variants: 3 on *APP*, 7 on *PSEN1*, 12 on *PSEN2*, 22 on MAPT, 16 on *GRN*, and 4 on *C9orf72*). Gene coordinates were derived from the GRCh38/hg38 human genome assembly and expanded by ±5 kilobases to include putative cis-regulatory elements. For each gene, all genotyped variants located within the extended region were extracted. Each score was also dichotomized at its median value, classifying subjects into “low” versus “high” genetic burden groups. Subjects were stratified by AD and FTD scores, creating four groups for each analysis: (i) AD and FTD subjects with low AD scores, (ii) AD and FTD subjects with high AD scores, (iii) AD and FTD subjects with low FTD scores, (iv) AD and FTD subjects with high FTD scores.

#### 4.4.3. Interaction Analysis

**Figure 4:**
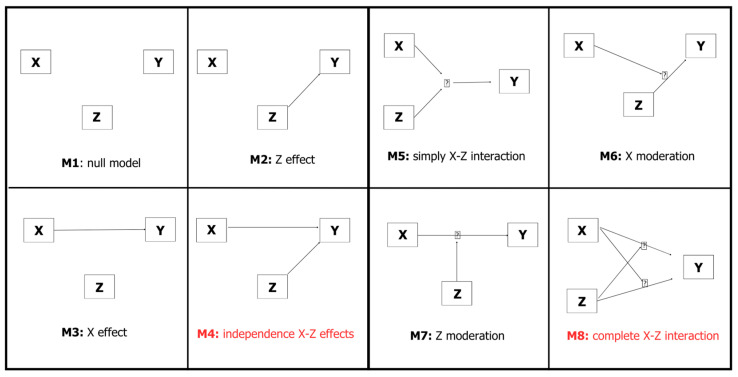
Seven different specifications tested using Fine–Gray models (plus the null model). M1–M4 are independence effects models while M5–M8 are interaction models. X represents the score of AD-related variants, Z represents the score of FTD-related variants, and Y denotes cumulative disease incidence. Models including both X–Z independence and complete interaction are highlighted in red, as these represent the model specifications of primary interest in our analysis.

Score stratification enabled comparison of cumulative incidence across diagnostic categories and levels of genetic burden, allowing the evaluation of whether a higher genetic load in one disease also influenced the cumulative incidence of the other. Cumulative incidence functions (CIFs) [42] were plotted to visually explore the relationship between diagnosis and onset, as well as the interaction effects between diagnosis and the dichotomized genetic scores on onset probability. Subsequently, the continuous AD and FTD scores were standardized and jointly evaluated in Fine–Gray models to assess their association with the cumulative incidence of disease onset under seven different specifications (see Figure 4): AD, FTD, AD+FTD, AD×FTD (interaction), AD+AD×FTD, FTD+AD×FTD, AD+FTD+AD×FTD. These models were fitted under two different cases (AD and FTD as the main event, respectively). The best-fitting model in each case was selected using the Akaike Information Criterion (AIC), and all models were adjusted for family clustering. To enhance robustness, all seven Fine–Gray models were re-estimated for both AD and FTD as the event of interest using 5-fold cross-validated genetic scores. The dataset was randomly partitioned into five approximately equal folds, stratified by diagnostic status (AD, FTD, or presymptomatic carriers) to ensure balanced representation across splits. For each iteration, variant effect estimates (β coefficients) were derived from four folds (training sets) using Fine–Gray competing risk models and subsequently used to compute AD and FTD genetic scores in the held-out fold (test set). This procedure ensured that score estimation for each individual was based exclusively on out-of-fold predictions. The fold-specific scores were then concatenated to obtain cross-validated genetic scores for all participants, which were subsequently used as independent variables in the Fine–Gray models. As an additional robustness check, all models were re-estimated after excluding carriers of the APOE ε4 allele.

#### 4.4.4. Statistical Software and Packages

All statistical analyses were conducted in R (version 4.3.2). Kaplan–Meier curves were generated and visualized using the “survival” and “survminer” packages, CIFs were plotted with the “cmprsk” package, and Fine–Gray competing risk regression models for clustered right censored data were implemented using the “crrSC” package. Linkage disequilibrium (LD) patterns were visualized using the “LDheatmap” package.

## 5. Conclusions

In conclusion, our findings suggest that polygenic modifiers may influence the course of monogenic AD. We observed that higher polygenic burden for one condition may be associated with lower incidence of the other, consistent with a context-dependent pattern of genetic interaction across neurodegenerative pathways. These results provide preliminary evidence of potentially informative genetic interplay between AD- and FTD-related backgrounds. Further studies in independent cohorts and mechanistic frameworks will be required to confirm these observations and clarify their biological relevance. These insights enrich our understanding of dementia biology and pave the way for more precise treatments in clinical practice.

## Figures and Tables

**Table 1:** Demographic and clinical characteristics of subjects included in the study stratified by diagnosis.

	Presymptomatic	AD	FTD	*p* Value
	(n = 107)	(n = 93)	(n = 226)	
Age at last follow-up, years	50.5	53.6	62.7	<0.0001 ^a^
	(15.2)	(14.4)	(9.8)	
Sex, F	54	40	103	0.55 ^b^
	(50.5%)	(43%)	(45.6%)	
Age at onset, years	/	51.1	59.9	<0.0001 ^a^
		(13.7)	(9.8)	

Mean (SD) for continuous variables, N (%) for categorical variables. ^a^ ANOVA test for continuous variables, ^b^ Chi-squared test for categorical variables.

**Table 2:** Demographic and clinical characteristics of subjects included in the study stratified by genetic groups.

	*APP*		*PSEN1*		*PSEN2*		*MAPT*		*GRN*		*C9orf72*	
	(n = 39)		(n = 71)		(n = 13)		(n = 29)		(n = 188)		(n = 86)	
	Pre Symptomatic	AD	Pre Symptomatic	AD	Pre Symptomatic	AD	Pre Symptomatic	FTD	Pre Symptomatic	FTD	Pre Symptomatic	FTD
	(n = 14)	(n = 25)	(n = 15)	(n = 56)	(n = 1)	(n = 12)	(n = 9)	(n = 20)	(n = 56)	(n = 132)	(n = 12)	(n = 74)
Age at last follow-up	52.9	64.6	37.6	46.1	60.0	66.2	60.9	50.7	51.3	64.8	51.5	62.1
	(14.0)	(12.8)	(9.6)	(10.8)	/	(8.2)	(20.2)	(12.7)	(14.0)	(8.7)	(16.0)	(8.4)
Sex, F	3	9	10	28	1	3	6	7	28	62	6	34
	(21.4%)	(36.0%)	(66.7%)	(50.0%)	(100%)	(25%)	(66.7%)	(35.0%)	(50.0%)	(47.0%)	(50.0%)	(46.0%)
Age at onset	/	62.4 (11.9)	/	44.0 (9.9)	/	60.7 (11.0)	/	47.4 (12.1)	/	62.4 (8.3)	/	57.0 (8.7)

Mean (SD) for continuous variables, N (%) for categorical variables. Age at last follow-up and age at onset are reported in years.

**Table 3:** Summary of the selected Fine–Gray models used to assess the individual and interactive effects of 5-fold cross-validated AD variants-related (x) and FTD variants-related (z) scores on the cumulative incidence of AD and FTD, respectively.

AD	HR (95% CI)	*p* Value	FTD	HR (95% CI)	*p* Value
x	2.49 (1.42–4.35)	0.0014	x	1.49 (0.997–2.21)	0.0515
z	1.82 (1.16–2.87)	0.0093	z	1.25 (1.07–1.46)	0.0051
x:z	0.21 (0.09–0.52)	0.0007			

## Data Availability

The data presented in this study are available in the Zenodo Data Repository at https://doi.org/10.5281/zenodo.18349665.

## Supplementary Materials

The following supporting information can be downloaded at: https://www.mdpi.com/article/10.3390/ijms27094081/s1.

## Abbreviations

The following abbreviations are used in this manuscript:

ACMG	American College of Medical Genetics and Genomics
AD	Alzheimer’s disease
AIC	Akaike Information Criterion
ANOVA	Analysis Of Variance
CIFs	Cumulative Incidence Functions
EOAD	Early Onset Alzheimer’s Disease
FTD	Frontotemporal dementia
GARDENIA	Genetic and epigenetic modulAtors in Rare neurodegenerative diseases with DEmentia: a National study on autosomal dominant Alzheimer disease and genetic frontotemporal degeneration with dementia
GENFI	Genetic FTD Initiative
GWAS	Genome-Wide Association Study
HGMD	Human Gene Mutation Database
HR	Hazard Ratio
LD	Linkage Disequilibrium
SD	Standard Deviation
sHRs	Subdistribution Hazard Ratios
VQSR	Variant Quality Score Recalibration
VUS	Variants of Uncertain Significance
