# Supplementary material for: Cumulative Incidence in Monogenic Alzheimer’s Disease and Frontotemporal Dementia: Gene–Gene Interaction Effect
Source: Int J Mol Sci. 2026 May 2;27(9):4081. doi: 10.3390/ijms27094081 (PMC13164024; doi:10.3390/ijms27094081)
Supplement: Supplementary file 1 [file ijms-27-04081-s001.zip › ijms-4213421-supplementary.pdf]

Table S1. OMIM (MIM) phenotype codes associated with the diseases linked to the genes analyzed in the study and corresponding inheritance patterns.

Gene	Phenotype	Phenotype OMIM number	Inheritance
APP	Alzheimer disease 1, familial	104300	AD
	Cerebral amyloid angiopathy, Dutch, Italian, Iowa, Flemish, Arctic variants	605714	AD
PSEN1	Alzheimer disease, type 3, with or without spastic paraparesis	607822	AD
	Dementia, frontotemporal	600274	AD
	Pick disease	172700	AD
PSEN2	Alzheimer disease-4	606889	AD
	Cardiomyopathy, dilated, 1V	613697	AD
MAPT	Frontotemporal dementia 1, with or without parkinsonism	600274	AD
	Pick disease	172700	AD
	Supranuclear palsy, progressive	601104	AD
	Supranuclear palsy, progressive atypical	260540	AR
GRN	Aphasia, primary progressive	607485	AD, AR
	Ceroid lipofuscinosis, neuronal, 11	614706	AR
	Frontotemporal dementia 2	607485	AD, AR
C9orf72	Frontotemporal dementia and/or amyotrophic lateral sclerosis 1	105550	AD

AD, Autosomal Dominant; AR, Autosomal Recessive

Table S2. List of *APP*, *PSEN1*, *PSEN2*, *MAPT*, *GRN*, and *C9orf72* causative variants of the subjects included in the study with coding nomenclature, number of carriers for each variant, variant classification, zygosity and ClinVar accession number where available.

Gene	Causative variant	Coding nomenclature	N. carriers (%)	Variant classification	n0	n1	n2	ClinVar accession number
APP	p.Ala713Thr	c.2137G>A	31 (79.5)	Likely pathogenic	3	28	395	VCV000018094.20
	p.Val717Ile	c.2149G>A	3 (7.7)	Pathogenic	0	3	423	VCV000018088.56
	p.Asp678Asn	c.2032G>A	2 (5.1)	Pathogenic	0	2	424	VCV000098236.1
	p.Thr719Pro	c.2155A>C	2 (5.1)	Pathogenic	0	2	424	VCV001342870.3
	p.Glu665Asp	c.1995G>C	1 (2.6)	VUS	0	1	425	VCV000018095.7
PSEN1	p.Met146Leu	c.436A>C	34 (47.9)	Pathogenic	0	34	392	VCV000018123.22
	p.Leu392Val	c.1174C>G	9 (12,7)	Pathogenic	0	9	417	VCV000098106.8
	p.Ile143Val	c.427A>G	6 (8.5)	Pathogenic	0	6	420	VCV002137603.4
	p.Cys92Ser	c.275G>C	3 (4.2)	Pathogenic	0	3	423	VCV000018142.1
	p.Met146Ile	c.438G>T	3 (4.2)	Pathogenic	0	3	423	VCV000098029.10
	p.Arg35Gln	c.104G>A	2 (2.8)	VUS	0	2	424	VCV000098004.19
	p.Leu174Met	c.520C>A	2 (2.8)	Pathogenic	0	2	424	VCV000018147.1
	p.Leu392Pro	c.1175T>C	2 (2.8)	Pathogenic	0	2	424	VCV000021026.8
	p.Val412Ile	c.1234G>A	2 (2.8)	VUS	0	2	424	VCV001318725.4
	p.Ala260Val	c.779C>T	1 (1.4)	Pathogenic	0	1	425	VCV000098075.4
	p.Asn135Asp	c.403A>G	1 (1.4)	Pathogenic	0	1	425	VCV000098021.9
	p.Glu184Gly	c.551A>G	1 (1.4)	Likely pathogenic	0	1	425	VCV000635001.6
	p.Glu363Gln	c.1087G>C	1 (1.4)	VUS	0	1	425	VCV002923936.3
	p.Met135Thr	c.404T>C	1 (1.4)	Pathogenic	0	1	425	VCV000098023.3
	p.Pro117Leu	c.350C>T	1 (1.4)	Pathogenic	0	1	425	VCV000098018.5
	p.Pro264Leu	c.791C>T	1 (1.4)	Pathogenic	0	1	425	VCV000098080.34
	p.Ser438Ala	c.1312T>G	1 (1.4)	Likely pathogenic	0	1	425	N/A
PSEN2	p.Met239Ile	c.717G>A	4 (30.8)	Pathogenic	0	4	422	VCV000008850.6
	p.Thr122Arg	c.365C>G	3 (23.1)	Pathogenic	0	3	423	VCV000008851.4
	p.Met239Val	c.715A>G	2 (15.4)	Pathogenic	0	2	424	VCV000008846.6
	p.Arg110Cys	c.328C>T	1 (7.7)	VUS	0	1	425	VCV002047817.4
	p.Gly212Glu	c.635G>A	1 (7.7)	Likely pathogenic	0	1	425	N/A
	p.Trp384Ter	c.1151G>A	1 (7.7)	VUS	0	1	425	N/A
	p.Val139Met	c.415G>A	1 (7.7)	VUS	0	1	425	VCV000448149.53
MAPT	p.Pro301Leu	c.902C>T	8 (27.6)	Pathogenic	0	8	418	VCV000014245.54
	IVS10+4 A>C	c.1920+4A>C	6 (20.7)	Pathogenic	0	6	420	N/A
	p.Val337Met	c.1009G>A	4 (13.8)	Pathogenic	0	4	422	VCV000014252.12

	IVS10+3 G>A	c.1920+3G>A	3 (10.3)	Pathogenic	0	3	423	N/A
	p.Gly16Val	c.47G>T	1 (3.4)	VUS	0	1	425	VCV000548576.15
	p.Gly335Ser	c.1003G>A	1 (3.4)	Pathogenic	0	1	425	VCV000098228.5
	p.Gly389Arg	c.1165G>A	1 (3.4)	Pathogenic	0	1	425	VCV000014255.10
	p.Pro569Leu	c.1706C>T	1 (3.4)	VUS	0	1	425	N/A
	p.Ser712Phe	c.2135C>T	1 (3.4)	Pathogenic	0	1	425	VCV000014262.4
	p.Val75Ala	c.224T>C	1 (3.4)	VUS	0	1	425	N/A
	p.Val287Ile	c.859G>A	1 (3.4)	VUS	0	1	425	VCV000962043.10
	p.Val755Ile	c.2263G>A	1 (3.4)	VUS	0	1	425	VCV000098231.47
	p.Leu271LeufsX10*	c.811_814del	142 (75.5)	Pathogenic	0	142	284	VCV000016020.46
	p.Cys139Arg	c.415T>C	11 (5.9)	VUS	0	11	415	VCV000589965.54
	p.Cys149LeufsX10	c.444_445del	5 (2.7)	Pathogenic	0	5	421	N/A
	p.Ala266Pro	c.796G>C	4 (2.1)	VUS	0	4	422	VCV001387314.11
	p.Gln341Ter	c.1021C>T	4 (2.1)	Pathogenic	0	4	422	N/A
	p.Thr382fs	c.1144dup	4 (2.1)	Pathogenic	0	4	422	VCV000098169.7
	p.Thr278SerfsX7	c.833_834del	3 (1.6)	Pathogenic	0	3	423	N/A
	p.Ala505Gly*	c.1514C>G	2 (1.1)	VUS	0	2	424	VCV001518387.8
	p.Arg418Gln	c.1253G>A	2 (1.1)	VUS	0	2	424	VCV000098178.26
	p.Cys126Trp	c.378C>G	2 (1.1)	VUS	0	2	424	N/A
GRN	c.709-3 C>G	c.709-3C>G	1 (0.5)	Pathogenic	0	1	425	VCV003899953.1
	p.Ala561Gly	c.1682_1683delinsGG	1 (0.5)	VUS	0	1	425	VCV002632289.2
	p.Arg110Ter	c.328C>T	1 (0.5)	Pathogenic	0	1	425	VCV000098134.55
	p.Arg564His	c.1691G>A	1 (0.5)	VUS	0	1	425	VCV001162824.9
	p.Asp22fs	c.63_64insC	1 (0.5)	Pathogenic	0	1	425	VCV000098121.1
	p.Cys157fs	c.468_474del	1 (0.5)	Pathogenic	0	1	425	VCV000098142.6
	p.Cys306Ter	c.918C>A	1 (0.5)	Pathogenic	0	1	425	VCV000807426.22
	p.Gln358Ter	c.1072C>T	1 (0.5)	Pathogenic	0	1	425	VCV000447470.9
	p.Met1Ter	c.2T>C	1 (0.5)	Pathogenic	0	1	425	VCV000016008.7
	p.Phe86SerfsX170	c.255del	1 (0.5)	Pathogenic	0	1	425	N/A
C9orf72	pathological expansion		86 (100)					

n0: number of homozygous minor allele; n1: number of heterozygotes; n2: number of homozygous major allele; N/A: not available. * One FTD patient is carrier of both mutations (Leu271LeufsX10 and Ala505Gly).

Table S3. Overview of non-causative variants identified by WES, including gene and chromosomal location, allele frequencies, genotype probabilities with zygosity, variant classification and ClinVar accession number where available.

Gene	Variant identified by WES	MAF	P.AA	P.AB	P.BB	n0	n1	n2	Variant classification	ClinVar accession number
APP	chr21:25982472	0	0	0.01	0.99	0	3	423	VUS	N/A
	chr21:26021842	0	0	0	1	0	1	425	VUS	N/A
	chr21:26112032	0	0	0	1	0	1	425	VUS	N/A
PSEN1	chr14:73192777	0	0	0	1	0	1	425	Likely pathogenic	N/A
	chr14:73192778	0	0	0	1	0	1	425	Likely pathogenic	N/A
	chr14:73192781	0	0	0	1	0	1	425	Likely pathogenic	N/A
	chr14:73192783	0	0	0	1	0	1	425	Likely pathogenic	N/A
	chr14:73192789	0	0	0	1	0	1	425	Likely pathogenic	N/A
	chr14:73192793	0	0	0	1	0	1	425	Likely pathogenic	RCV001808318.5
	chr14:73206470	0.02	0	0.04	0.96	0	15	411	Benign	RCV000559984.12
PSEN2	chr1:226883729	0	0	0	1	0	1	425	Likely benign	RCV000172098.12
	chr1:226883748	0.01	0	0.02	0.98	0	7	419	Benign/Likely benign	RCV000641029.18
	chr1:226883799	0	0	0	1	0	1	425	VUS	RCV005099416.2
	chr1:226883912	0	0	0	1	0	1	425	VUS	N/A
	chr1:226885596	0	0	0	1	0	2	424	VUS	RCV006452703.1
	chr1:226888091	0	0	0	1	0	1	425	VUS	N/A
	chr1:226888112	0	0	0.01	0.99	0	3	423	Benign/Likely benign	RCV001664491.5
	chr1:226889038	0	0	0	1	0	1	425	VUS	N/A
	chr1:226889049	0	0	0	1	0	1	425	Likely pathogenic	N/A
	chr1:226890127	0	0	0	1	0	1	425	VUS	RCV003620737.4
	chr1:226890129	0	0	0	1	0	1	425	VUS	N/A
	chr1:226890131	0	0	0	1	0	1	425	Likely pathogenic	N/A
MAPT	chr17:45983126	0.05	0	0.10	0.90	0	43	383	Benign	RCV001721974.3
	chr17:45983252	0	0	0	1	0	1	425	VUS	N/A
	chr17:45983253	0	0	0	1	0	2	424	VUS	N/A
	chr17:45983409	0.34	0.11	0.46	0.43	46	198	182	Benign	RCV001510735.9
	chr17:45983468	0	0	0.01	0.99	0	3	423	Likely benign	RCV003947981.2
	chr17:45983475	0	0	0.01	0.99	0	3	423	Benign	RCV000761992.39
	chr17:45983493	0.06	0	0.12	0.88	1	50	375	Benign	RCV000555583.12
	chr17:45983657	0.35	0.11	0.48	0.41	46	205	175	Benign	RCV001510736.9
	chr17:45983670	0.35	0.11	0.48	0.41	46	205	175	Benign	RCV001510737.9
	chr17:45983757	0	0	0	1	0	2	424	Benign	RCV001517496.10
	chr17:45983912	0.34	0.11	0.47	0.42	46	200	180	Benign	RCV000989933.10
	chr17:45989975	0	0	0.01	0.99	0	3	423	Benign/Likely benign	RCV000585116.39
	chr17:45990016	0.16	0.01	0.30	0.69	5	126	295	Benign	RCV001514001.10
	chr17:45990034	0.35	0.11	0.47	0.42	48	200	178	Benign	RCV000989935.10
	chr17:45999299	0.35	0.11	0.47	0.42	47	202	177	Benign	N/A
	chr17:45999344	0	0	0	1	0	1	425	Benign	RCV004080981.1
	chr17:46025124	0	0	0	1	0	1	425	VUS	N/A
	chr17:46031496	0	0	0	1	0	1	425	VUS	N/A
	chr17:46031525	0	0	0	1	0	1	425	VUS	N/A
	chr17:46031540	0.35	0.11	0.48	0.42	46	203	177	Benign	RCV001510742.9
	chr17:46031648	0	0	0	1	0	2	424	VUS	RCV001063200.10
	chr17:46032108	0.36	0.12	0.47	0.41	52	199	175	Benign	RCV000989938.11
GRN	chr17:44349274	0	0	0	1	0	2	424	VUS	N/A
	chr17:44350237	0.01	0	0.02	0.98	0	7	419	Benign/Likely benign	N/A
	chr17:44350299	0	0	0	1	0	1	425	VUS	VCV000098140.30
	chr17:44350732	0	0	0	1	0	1	425	VUS	RCV003076593.5

	chr17:44352751	0	0	0	1	0	1	425	VUS	RCV001938835.9
	chr17:44354055	0	0	0	1	0	1	425	VUS	N/A
	chr17:44354062	0	0	0	1	0	1	425	VUS	N/A
	chr17:44354064	0	0	0	1	0	1	425	VUS	N/A
	chr17:44354074	0	0	0	1	0	1	425	VUS	N/A
	chr17:44354263	0	0	0	1	0	1	425	VUS	N/A
	chr17:44354425	0	0	0	1	0	1	425	VUS	N/A
	chr17:44354426	0	0	0	1	0	1	425	VUS	N/A
	chr17:44354427	0	0	0	1	0	1	425	VUS	N/A
	chr17:44354533	0	0	0	1	0	1	425	VUS	RCV005341449.1
	chr17:44354667	0	0	0	1	0	1	425	VUS	RCV005559419.1
	chr17:44356183	0	0	0	1	0	1	425	VUS	N/A
C9orf72	chr9:27556720	0	0	0	1	0	1	425	VUS	N/A
	chr9:27561630	0.11	0.01	0.19	0.80	4	82	340	Benign	RCV000606644.5
	chr9:27561642	0	0	0	1	0	1	425	VUS	N/A
	chr9:27566975	0	0	0.01	0.99	0	3	423	VUS	N/A

MAF: Minor Allele Frequency; P.AA, P.AB, P.BB: genotypes probability; n0: number of homozygous minor allele; n1: number of heterozygotes; n2: number of homozygous major allele; N/A: not available.

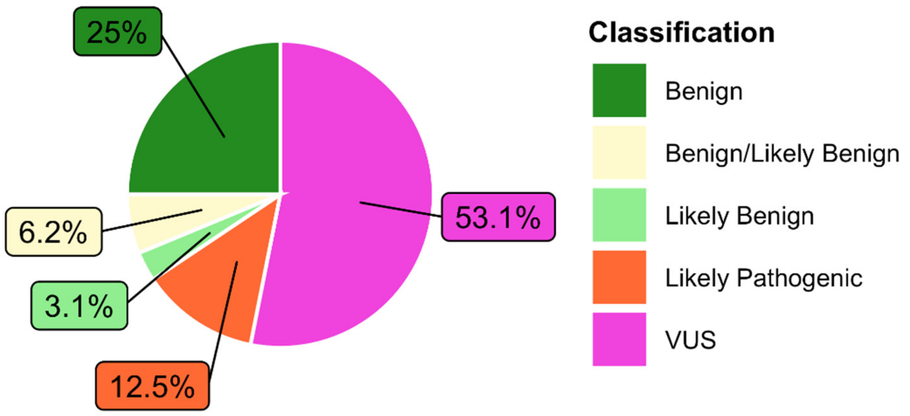

Figure S1. Non-causative variants classification. Pie chart showing the distribution of the non-causative variants identified in *APP*, *PSEN1*, *PSEN2*, *MAPT*, *GRN*, and *C9orf72*.

Table S4. Log-likelihood (logL), number of parameters (T), and Akaike Information Criterion (AIC) for the seven Fine–Gray model specifications with AD or FTD as event of interest and raw genetic scores as independent variables. The full interaction model (x+z+x:z) was retained due to comparable AIC values with best-fitting models and a statistically significant interaction term. Best-fitting models (minimum AIC) are shown in bold, and selected interaction models in bold italics. Hazard ratios (HR), 95% confidence intervals (CI), and p-values are reported for the selected interaction models, describing the effects of AD-related (x) and FTD-related (z) genetic burden scores and their interaction on cumulative incidence.

**AD**	logL	T	AIC	**FTD**	logL	T	AIC
x	-525.471	1	1052.942	x	-1209.644	1	2421.288
z	-528.028	1	1058.055	**z**	**-1202.512**	**1**	**2407.025**
**x+z**	**-520.400**	**2**	**1044.799**	**x+z**	**-1187.144**	**2**	**2406.441**
x:z	-531.301	1	1064.601	x:z	-1210.476	1	2422.952
x+x:z	-525.350	2	1054.699	x+x:z	-1209.017	2	2422.034
z+x:z	-527.186	2	1058.372	z+x:z	-1202.385	2	2408.771
x+z+x:z	***-520.031***	***3***	***1046.063***	***x+z+x:z***	***-1201.103***	***3***	***2408.206***
**AD**	HR (95% CI)	*p* value		**FTD**	HR (95% CI)	*p* value	
x	2.84 (2.05–3.93)	3.74×10^{-10}		x	1.25 (1.18–1.33)	2.19×10^{-12}	
z	1.79 (1.22–2.62)	2.84×10^{-3}		z	1.32 (1.22–1.43)	3.94×10^{-12}	
x:z	0.68 (0.52–0.88)	3.87×10^{-3}		x:z	1.06 (1.02–1.10)	6.23×10^{-3}	

Table S5. Log-likelihood, number of predictors (T), and AIC for the seven Fine–Gray models tested, with AD or FTD as the event of interest and 5-fold cross-validated genetic scores as independent variables. The full interaction model was selected for AD and the full independent effects model (x+z) was selected for FTD based on AIC. Best-fitting models are shown in bold.

**AD**	logL	T	AIC	**FTD**	logL	T	AIC
x	-532.5096	1	1067.019	x	-1210.863	1	2423.727
z	-531.5713	1	1065.143	z	-1209.597	1	2421.194
x+z	-530.5662	2	1065.132	**x+z**	**-1207.842**	**2**	**2419.683**
x:z	-532.4051	1	1066.81	x:z	-1210.621	1	2423.242
x+x:z	-530.4429	2	1064.886	x+x:z	-1210.618	2	2425.236
z+x:z	-530.1842	2	1064.368	z+x:z	-1209.415	2	2422.829
**x+z+x:z**	**-527.5896**	**3**	**1061.179**	x+z+x:z	-1207.525	3	2421.05

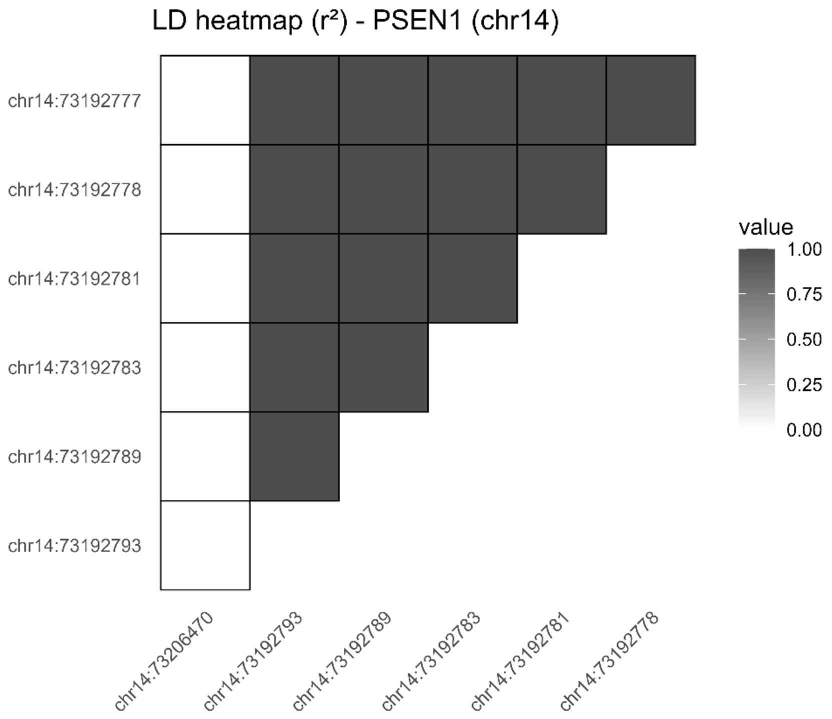

Figure S2. Linkage Disequilibrium heatmap. LD heatmap showing LD in *PSEN1*.
